# Typhoid Fever

**Published:** 1893-11

**Authors:** William B. Conway

**Affiliations:** Athens, Ga.


### TYPHOID FEVER.

By WILLIAM B. CONWAY, M. D.,
ATHENS, GA.

Those who are particularly interested in infectious diseases, and knowing full well the dangers accompanying them, are deeply impressed with a feeling that we are nearing the time when discoveries of extraordinary importance may be made at any time in the practice of medicine, such as we have seen in the last few years in surgery. The results of hard work of many of our modern experimenters have had a tendency to bring us nearer and nearer every day towards a definite knowledge of the methods by which the human system fights against disease, and its protection against toxic influences. Not that I have anything new to present to you in this article, but should I throw any light on the subject I feel that I am fully compensated.

No section of the world is exempt from the ravages of typhoid fever, and every medical man is naturally interested in anything tending to lessen the dangers of this disease. Most of our writers are of the opinion that it is caused by the introduction into the system of bacteria, conveyed there by impure water, milk, or by atmospheric influences. A specific germ ; but no antidote has been discovered for its cure. Typhoid fever is always to be regarded as a serious disease. Among children it is milder and of shorter duration. Some cases are so slight that they may be termed walking cases.

Complications render cases unfavorable or hopeless, according to their character. Dr. Page, of New York, says: "Under the ordinary expectant plan of treatment in the city about forty-two per cent. of cases die." This specific poison acts by destroying tissue and reducing vital power, producing an abnormal amount of heat, accompanied by nervous, cardiac and bowel symptoms, such as are seen in sunstroke and heat exhaustion, thereby depressing all the vital functions. In a strong, robust person the temperature may rise suddenly to 106° F., but this is not an indication of any very great danger, but when we meet with one whose temperature is 103° F., and remains so day after day, we become apprehensive ; we know it does mean danger, and of course we are watchful and careful of its results. Lesions of the intestinal and mesenteric glands, enlargement of the spleen and muscular degeneration take place.

During the first week the small intestines are irritable, congested and painful upon palpation, and unless resolution occurs, ulceration of Peyer's patches and the mesenteric glands set in during the second and third week and perforation may occur at any time.

The heart, according to Zinker, undergoes temporary waxy degeneration, and hence this important fact should be noted and cardiac depressants withheld. Tympanites, caused by an accumulation of gas, is a frequent symptom, but not always present. Diarrhea usually occurs during the first week ; appearing later on is a much more serious symptom. Delirium may appear at any time, so also dullness of intellect, twitching of muscles and insomnia. The tongue, at first covered with a thin white coat, becomes later on red and sometimes fissured. After an experience of over twenty years practice in the county of Montgomery, Virginia, upon the summit of the Alleghany mountains, I find that complications are more frequent in the number of cases treated by me in Athens, Georgia.

Temperature—is there any special danger from it? It is said that leucocytes die in a temperature of 108° F. So does the patient (?), notwithstanding Duckworth reports a case of a woman twenty-six years of age in whom the thermometer is stated to have registered a temperature of 228° F.

Smart says: "Animal temperature is not a reliable index to tissue change; that it is by no means a certain indication of the gravity of the disease. German writers are divided, and we see in much of our American medical literature of to-day the same sentiment. Part absolutely deny the influence of treatment on the mortality of fever, which reminds us of what some of us have heretofore depended upon, and still depend upon, that is, on the *vis medicatrix naturæ.* The coal tar preparations have been used extensively during the past few years, and are still relied upon by many of the profession as decreasing heat production, as well as increasing heat dissipation.

Childs reports a case of toxic effects of acetanilid in twenty-five, fifteen and ten grains, respectively, within twenty-four hours. Symptoms, cyanosis, syncope, subnormal temperature and excitement. Pyrexia is in some degree to be considered a conservative process not to be checked. The mere control of the temperature, without looking carefully into the existing conditions, is not productive of good, but often of harm; and the use of antipyretics, if given at all, should be at short periods, and only then in selected cases. They may do good primarily, but the effects upon the nervous system, and especially the heart's action, multiply its direct action upon the fever, and irreparable damage is the result.

As to the treatment of typhoid fever the profession is divided, each having his especial line of treatment, basing it, as he should do, upon what he considers the pathology of the disease, and using just such line of remedies as has served him best in his own enlightened judgment. This is natural, as there is no specific for typhoid fever, but each case has to be conducted according to the conditions present. In those who are accustomed to use of intoxicants and delicate, broken down females stimulants are called for from the start; the same case with opiates when the patient is accustomed to their use. The trouble is, we seldom see a patient in the incipient stage; nearly all the cases steal in, as it were, upon him: headache, pain in the back and limbs, loss of appetite, general malaise, followed for several days by general indisposition, not sick enough to go to bed, nor well enough to attend to his duties. A dose of castor oil and turpentine, a C.C. pill or a patent medicine pill of some kind has been taken by him, and finding that nothing has done good, but, on the contrary, he has steadily grown worse, he takes his bed and consults a physician. In such a case we would likely use no purgative. I would suggest a warm bath, cleansing the skin thoroughly, a dose of phenacetine or antikamnia to relieve headache, and the free use of spirits mindereri and acetate of potash for twenty-four hours, in order to arouse the action of the skin and kidneys; in most cases a free diaphoresis is very essential, and if we can accomplish that object without the use of antipyretics it leaves the system in a much better condition to withstand the ravages of the disease. After this sulphate quinine and salol may be given for several days, especially if any malarial trouble is suspected. Sponging with cold water during the morning and evening rise of temperature, especially if the temperature exceeds 103° F. One-fourth of a grain of sulphate morphia before bedtime gives refreshing sleep; if morphine is contraindicated use the bromides. Digitalis and brandy may be given should the heart's action become feeble or irregular.

The time-honored turpentine emulsion should be relegated to the rear along with aconite and veratrum viride, if Dr. Page is correct in his statement. He says "turpentine internally is worthless if not dangerous, and externally, in the shape of stupes, it is disagreeable if not absolutely useless." He says, also: "The object is not the administration of any particular antidote to the virus, but rather to sustain life until the poison has been eliminated." Why then should we use local applications in diphtheria and other infectious diseases? If so, should it not require a most persistent antiseptic treatment in order to destroy the bacteria or prevent their increase and hasten the absorption of the poison which they produce? In my opinion the point is to keep antiseptics long enough in direct contact with the diseased glands along the tract of the lower bowels so as to completely destroy the virus. Following up this line of reasoning brings to my mind an article I noticed some time ago published in the *Virginia Medical Monthly*, and written by Dr. Bedford Brown of Alexandria, Va., on intestinal septic infection in typhoid fever. This treatment I have used in a number of cases with the happiest effects, and can heartily indorse it. I hope to hear more on the subject. It is this: give one grain of bisulphide of calcium every three hours, also,

℞. Iodoform, } aa ʒ ss.

Creosote, }

M.—Capsules No. xxx. Sig.: One every three hours.

If diarrhoea is present a small quantity of opium is added, and if hemorrhage occurs, use the following:

℞. Iodoform, ℈ i.

Creosote, gtts. xx.

Tannin, ℈ ii.

Opii. pulv., grs. v.

Ergotini, ℈ iss.

M.—Capsules No. xx. Sig.: One capsule every hour or two.

I am sure that I have seen excellent results from the use of these drugs in the intestinal canal, because of the prevention of further putrefaction and fermentation. The diet is, of course, another very important matter, and should be carefully looked after—milk, meat broth, rice water, raw eggs and brandy or whisky.

